# Current status of periodontal disease in adults in Takahagi, Japan: a cross-sectional study

**DOI:** 10.1186/s12903-020-1046-4

**Published:** 2020-02-19

**Authors:** Satoshi Sekino, Ryoichi Takahashi, Yukihiro Numabe, Hiroshi Okamoto

**Affiliations:** 1 Nippon Dental University, School of Life Dentistry, Department of Periodontology, 1-9-20, Fujimi, Chiyodaku, Tokyo, Japan; 2 Tokyo Periodontal Treatment Center, 16-14, Koamichio, Nihonbashi, Chuo-ku, Tokyo, Japan

**Keywords:** Periodontal disease, Prevalence, Extent, Severity, Japanese population

## Abstract

**Background:**

To date, a few studies have documented the detailed periodontal conditions of a Japanese population. It is important to know if the awareness of Japanese nationals and dentists regarding oral hygiene and prevention of periodontal disease have improved when compared with the past in Japan for the development of future scenarios regarding prevention. The aim of this study was to investigate the severity, prevalence, and extent of periodontal disease in the adult population of the city of Takahagi, Japan. Results were also compared with those of an epidemiological study performed in Japan in the 1980s.

**Methods:**

A total of 582 (aged 20 to 89 years) randomly sampled Takahagi residents answered a comprehensive questionnaire and participated in clinical examinations.

**Results:**

The mean percentages of tooth surfaces harboring plaque and exhibiting BOP were 59.5 ± 20.9% and 31.1 ± 21.1%, respectively. The mean PPD and CAL were 2.5 ± 0.5 mm and 2.9 ± 1.0 mm, respectively. Compared with results of the 1980s survey, the mean percentages of plaque and bleeding on probing were lower in the current population. The mean CAL and prevalence of attachment loss of ≥5 mm in some age groups were higher in the present study than in the 1980s study. There were no statistically significant differences with respect to mean probing depth between the 1980s and current age groups.

**Conclusions:**

Periodontal disease was still prevalent in the current Japanese population, even though some improvement occurred. Proper public health programs therefore need to be established.

## Background

Periodontitis is a progressive, chronic inflammatory disease. Its clinical symptoms include increased probing pocket depth, attachment loss, bleeding on probing, suppuration, and loosening of teeth, leading eventually to tooth loss.

Global epidemiological studies have found that periodontitis is extremely common [1–3]. Recent studies have also found a high prevalence of periodontitis in developing countries and among poor and minority populations [4–8]. In Western nations, however, periodontitis is less of a problem than it was 20 to 30 years ago [9–11].

In Japan, the National Survey of Dental Diseases in 2011 [12] found that 13.5 to 46.2% of individuals 20 to 59 years of age had periodontal pockets ≥4 mm in depth, a lower prevalence than that reported in 1999 and 2005. As a result of more people keeping larger numbers of their own teeth, however, the proportion of people over 65 years of age with pockets ≥4 mm had increased compared with the proportion 12 years before. That survey, however, covered probing depth of only ten teeth and did not include a detailed analysis of clinical parameters such as attachment level.

An epidemiological survey including full mouth periodontal examinations was carried out in 1985 and 1986 in Ushiku City, Ibaraki Prefecture, Japan [13, 14]. Ushiku City is located approximately 50 km from suburban Tokyo. At the time, Ushiku City had a population of 50,000, the percentage of the population over 65 years old was about 7% and that under 15 years old was about 27%. They had around 20 to 25 dental clinics, mainly performing symptomatic therapy. The study found that the mean plaque score and gingival bleeding rate of the residents were 64 and 48%, respectively, and that the degree of attachment loss increased with age. To date, the Ushiku study is the only one to document the detailed periodontal conditions of a Japanese population. It is important to know if the awareness of Japanese nationals and dentists regarding oral hygiene and prevention of periodontal disease have improved when compared with the past in the same district in Japan for the development of future scenarios regarding prevention.

The objective of this study was to survey the severity, prevalence, and extent of periodontal disease in adult residents of Takahagi City, Ibaraki Prefecture, Japan. We also compared the results of this survey with those of an epidemiological survey of the same part of Japan in the 1980s.

## Methods

**Fig. 1:**
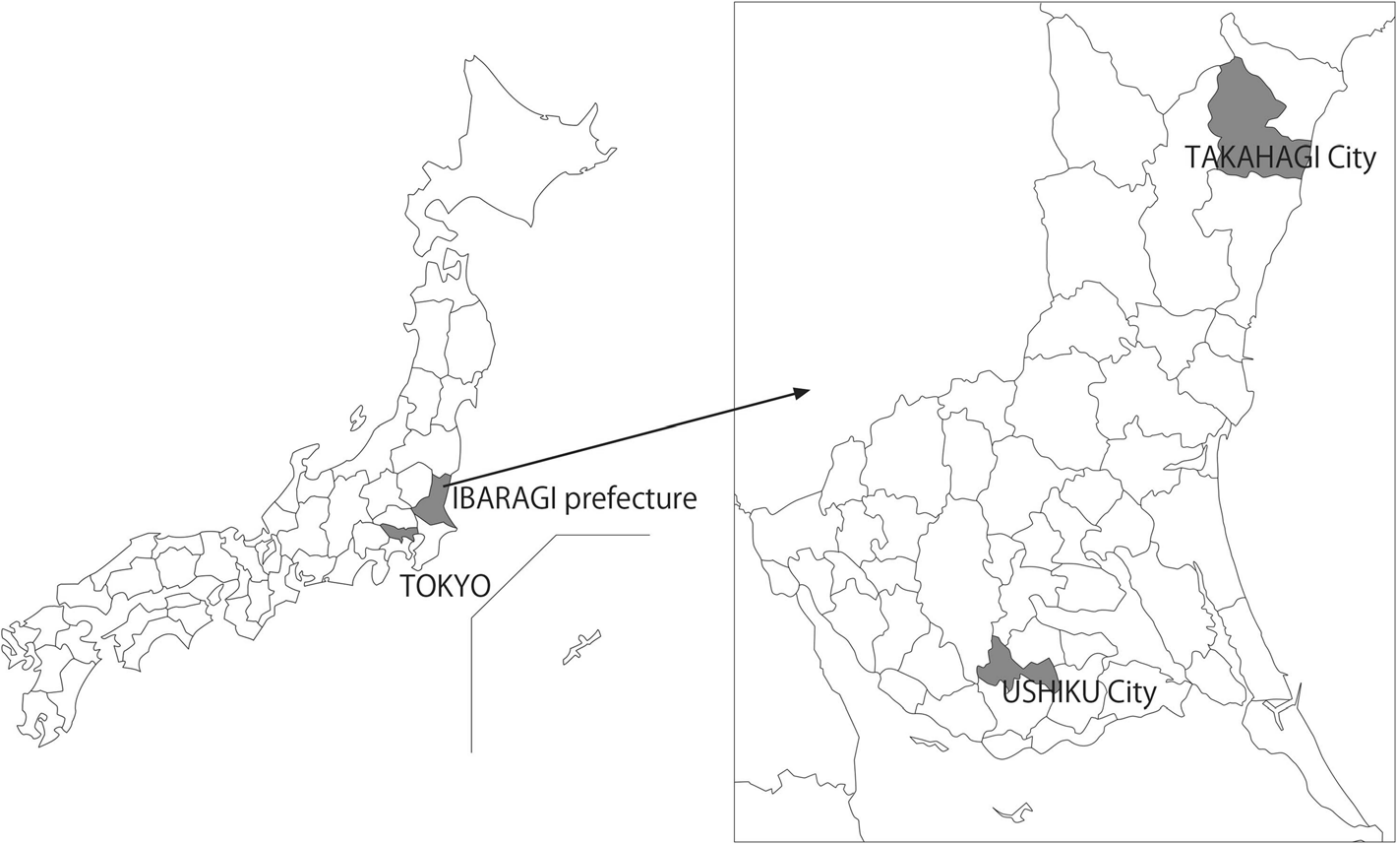
The map of the city of Takahagi and Ushiku in Ibaragi prefecture, Japan

The survey participants were residents of Takahagi City, Ibaraki Prefecture, aged between 20 and 89 years. Takahagi City has a population of around 30,000 and is located approximately 200 km from Tokyo (Fig. 1). The percentage of the population over 65 years old is about 30% and that under 15 years old is about 11%. It is in the same prefecture as Ushiku City. Its population, population density, and area make it a moderately sized regional Japanese city. There are 15 dental clinics. The municipal area includes both urban and rural localities, and the labor force includes both blue- and white-collar workers; in Japanese terms, its economy is also medium in size.

We randomly sampled approximately 1400 inhabitants from individuals living in Takahagi City, and 582 answered a comprehensive questionnaire and participated in clinical examinations. The clinical investigation was carried out by an experienced periodontist (S.S.) in a dental examination room within Takahagi City Hall between April 2010 and March 2013. All people who agreed to take part became participants after receiving an explanation of periodontal disease and the examinations included. Subsequently, they completed a questionnaire about their characteristics, including general medical history, history of smoking, height, weight, oral hygiene habits, history of regular dental checkups, history of periodontal treatment, and subjective symptoms. After the mouth had been checked for matters such as missing teeth, caries, tooth restoration, and malalignment, the clinical parameters described below were recorded for all teeth, including the third molars. This study was approved by the Institutional Review Board of Nippon Dental University. The Nippon Dental University made an agreement for cooperation in epidemiological research with Takahagi City in 2012.

### Plaque adhesion

Plaque adhesion was assessed by visual examination and scraping with a probe [15].

### Probing pocket depth (PPD)

Distance from the gingival margin to the bottom of the pocket was measured to the nearest millimeter with a pressure-sensitive probe. The probing force was 25 g and the tip diameter was 0.5 mm (TUCL probe®, Shioda, Tochigi, Japan). 

### Clinical attachment level (CAL)

The distance from the cement–enamel junction to the bottom of the pocket was measured to the nearest millimeter with a pressure-sensitive probe, as described above.

### Bleeding on probing (BOP)

The presence or absence of bleeding from the bottom of pockets after probing was checked with a pressure-sensitive probe, as described above. BOP was considered positive if bleeding occurred within 10 s after probing.

### Recorded sites

Plaque adhesion was recorded for four surfaces (buccal, lingual, mesiobuccal, and distobuccal) of each tooth. BOP, PPD, and CAL were recorded for six surfaces, the four previously mentioned plus the mesiolingual and distolingual surfaces.

### Reproducibility of measurements

In order to check for intra-operator reproducibility of measurements, five volunteers underwent whole-jaw probing, with PPD and CAL measured twice. The κ coefficient was 0.93 for PPD measurements and 0.86 for CAL.

### Data analysis

Characteristics of study subjects were expressed as numbers (percentages) or means and standard deviation (SD) or standard error (SE), and were stratified by age. Prevalence of at least one affected site and the extent (proportion) of affected sites on teeth per mouth by degree of clinical attachment level (CAL, cut-offs at ≥3 mm and ≥ 5 mm,) or probing pocket depth (PPD, cut-offs at ≥4 mm and ≥ 6 mm) and the mean values of all clinical parameters were calculated for all participants and for each age group from 20 to 34 years to more than 75 years. The cumulative percentile plots of CAL ≥3 mm and ≥ 5 mm were calculated for all subjects.

Differences in characteristics of the subjects and clinical parameters between age groups were evaluated by chi squared test or one-way analysis of variance.

The mean percentages of CAL ≥5 mm in each age group of different demographic, biologic, environmental parameters were calculated and compared using Student’s t-test or Welch’s t test.

## Results

Five hundred and eighty-two participants were included in the study. The mean age of study subjects was 55.3 ± 16.4 years. The mean proportion of tooth surfaces harboring plaque was 59.5%, and was highest for participants in the oldest age group and lowest for those aged 55–64 years. Detailed information about the characteristics of the participants is presented in Table 1.

**Table 1:** Characteristics of study subjects with periodontal examinations

	Age Groups (years)							
	20–34	35–44	45–54	55–64	65–74	75+	*P* Vallue	Total
Number of subjects	96	72	61	132	177	44		582
Age,years	28.6 ± 3.8	39.0 ± 2.7	49.3 ± 2.9	59.6 ± 2.7	69.3 ± 2.7	78.8 ± 3.4		55.3 ± 16.4
Male gender(%)	27 (28.1)	28 (39.4)	17 (27.8)	31 (23.4)	73 (41.2)	16 (36.4)	< 0..05^*^	33
Smoking status								
Never smokers(%)	64 (66.7)	53 (73.6)	42 (68.9)	104 (78.8)	125 (70.6)	28 (63.6)	< 0..01^*^	416 (71.5)
Former smokers(%)	21 (21.9)	10 (13.9)	12 (19.7)	17 (12.9)	48 (27.1)	16 (36.4)		124 (21.3)
Current smokers(%)	11 (11.5)	9 (12.5)	7 (11.5)	11 (8.3)	4 (2.3)	0 (0)		42 (7.2)
Educational level								
Low (%)	0 (0)	10 (13.9)	0 (0)	17 (12.9)	49 (27.7)	18 (41.0)	< 0..01^*^	94 (16.2)
Middle(%)	32 (33.3)	26 (36.)	29 (47.5)	82 (62.1)	102 (57.6)	22 (50)		293 (50.3)
High(%)	64 (66.6)	36 (50)	32 (52.5)	33 (25)	26 (14.7)	4 (9.1)		195 (33.5)
Body Mass index								
< 25 kg/m^2^	83 (86.5)	64 (88.9)	49 (80.3)	107 (81.1)	136 (76.8)	33 (75)	< 0..01^*^	382 (65.6)
25–30 kg/m^2^	11 (11.5)	7 (9.7)	12 (9.7)	22 (16.7)	37 (20.9)	11 (25)		99 (17.0)
> 30 kg/m^2^	2 (2.1)	1 (1.4)	0 (0)	3 (2.3)	4 (2.3)	0 (0)		10 (1.7)
Diabetes Melitus	0 (0)	0 (0)	1 (1.6)	7 (5.3)	13 (7.3)	5 (11.4)	< 0..05^*^	
Tooth brushing frequency								
< 2 times/day	5 (5.2)	9 (12.5)	10 (16.4)	8 (6.1)	32 (18.1)	9 (20.50)	0.072^*^	73 (12.5)
≥ 2 times/day	91 (94.8)	63 (87.5)	51 (83.6)	124 (93.9)	145 (81.9)	35 (79.5)		509 (87.5)
Use of inter-dental care device								
No	70 (72.9)	43 (59.7)	33 (54.1)	68 (51.5)	84 (47.5)	25 (56.8)	< 0..01^*^	323 (55.5)
Yes	26 (27.1)	29 (40.3)	28 (45.9)	64 (48.5)	93 (52.5)	19 (43.)		259 (44.5
Last dental visit								
Within last 12 months	20 (20.8)	16 (22.2)	19 (31.1)	52 (39.4)	83 (46.9)	15 (34.1)	< 0..01^*^	205 (35.2)
Less often	76 (79.2)	56 (77.8)	42 (68.9)	80 (60.6)	94 (51.3)	29 (65.9)		377 (64.8)
Tooth count in dentates*(excluding third molars)	27.7 ± 0.8	27.0 ± 1.9	26.0 ± 3.0	24.9 ± 3.8	23.1 ± 5.3	17.2 ± 8.4	< 0.001^†^	24.6 ± 5.1
Tooth count in dentates	28.5 ± 1.5	28.0 ± 1.4	27.2 ± 2.9	26.0 ± 3.6	24.6 ± 4.7	17.8 ± 7.3	< 0.001^†^	25.7 ± 4.7
Plaque score	63.4 ± 17.7	56.5 ± 20.3	57.4 ± 20.0	54.1 ± 18.9	57.4 ± 20.8	78.6 ± 19.5	< 0.001^†^	59.5 ± 20.9

Data are presentred as numvers (percentages) or means ±standard deviaions (SD)

^*^P value for differences between groups using the ×^2^ test

^†^*P* value for differences between groups using the one-way ANOVA

Table 2 shows the periodontal parameters of this sample according to age. BOP was highest for participants in the oldest age group and lowest for those aged 35–44 years. The mean values for both PPD and CAL were lowest among participants in the youngest group and increased with advancing age. The prevalence of participants with CAL ≥3 mm accounted for 100%, except for the young age groups. Those with CAL ≥5 mm accounted for only 22.9% of participants in the youngest age group, but for 98.8% in the oldest age group. The extent of attachment loss became broader with advancing age. As with CAL, the proportion of participants with a low maximum value of PPD was greatest in the younger age groups and decreased in older age groups, and the proportion of participants with the PPD ≥6 mm was highest in the older age groups.

**Table 2:** Prevalence, extent and mean values of each periodontal parameter

	Age Groups (years)							
	20–34	35–44	45–54	55–64	65–75	75+	P Value	Total
CAL measures								
Prevalence CAL ≥3 mm (%)	89.6 (3.1)	98.6 (10.6)	100 (0)	100 (0)	100 (0)	100 (0)	< 0.001^*^	98.2 (0.6)
Prevalence CAL ≥5 mm (%)	22.9 (4.3)	47.2 (5.9)	65.6 (6.1)	82.6 (3.3)	91.5 (2.1)	98.8 (1.6)	< 0.001^*^	70.5 (1.9)
Proportion of sites/mouth CAL ≥3 mm (%)	18.6 (2.0)	40.0 (2.4)	50.4 (2.7)	56.3 (1.6)	67.3 (1.5)	75.0 (1.7)	< 0.001^†^	51.6 (1.1)
Proportion of sites/mouth CAL ≥5 mm (%)	0.8 (0.2)	3.2 (0.7)	7.7 (2.2)	9.9 (1.2)	19.7 (1.5)	21.8 (2.6)	< 0.001^†^	11.0 (0.7)
Proportion of teeth/mouth CAL ≥3 mm (%)	48.9 (3.4)	80.9 (2.4)	88.4 (1.9)	92.8 (0.8)	95.8 (0.6)	98.4 (0.6)	< 0.001^†^	84.3 (1.0)
Proportion of teeth/mouth CAL ≥5 mm (%)	3.9 (0.8)	11.0 (1.9)	17.9 (3.1)	27.3 (2.2)	44.7 (2.2)	49.9 (3.9)	< 0.001^†^	26.9 (1.2)
Mean CAL (mm)	1.8 (0.1)	2.4 (0.1)	2.8 (0.1)	3.0 (0.1)	3.5 (0.1)	3.6 (1.0)	< 0.001^†^	2.9 (0.04)
PPD measures								
Prevalence PPD ≥4 mm (%)	79.1 (4.1)	88.7 (8.1)	82.3 (4.9)	89.5 (2.7)	96.1 (1.5)	97 (2.6)	< 0.001^*^	90.3 (1.2)
Prevalence PPD ≥6 mm (%)	20.8 (4.1)	35.2 (5.6)	32.3 (6.0)	42.5 (4.3)	57.9 (3.7)	63.6 (7.3)	< 0.001^*^	43.1 (2.1)
Proportion of sites/mouth PPD ≥4 mm (%)	4.2 (0.6)	6.3 (1.0)	7.7 (1.5)	9.1 (1.0)	14.0 (1.1)	20.0 (2.3)	< 0.001^†^	10.0 (0.5)
Proportion of sites/mouth PPD ≥6 mm (%)	0.2 (0.1)	1.0 (0.3)	2.1 (0.8)	2.2 (0.5)	3.4 (0.5)	5.0 (1.3)	< 0.001^†^	2.3 (0.2)
Proportion of teeth/mouth PPD ≥4 mm (%)	16.9 (1.8)	21.8 (2.5)	22.8 (2.8)	24.9 (1.7)	31.1 (1.8)	29.4 (2.8)	< 0.001^†^	25.1 (0.9)
Proportion of teeth/mouth PPD ≥6 mm (%)	1.4 (0.3)	4.3 (1.0)	5.9 (1.7)	6.1 (1.0)	8.7 (0.9)	9.2 (1.4)	< 0.001^†^	6.1 (0.5)
Mean PPD (mm)	2.3 (0.02)	2.4 (0.03)	2.5 (0.1)	2.5 (0.04)	2.6 (0.05)	2.9 (0.1)	< 0.001^†^	2.5 (0.02)
Bleeding on probing (%)	32.1 (1.6)	27.9 (1.7)	30.9 (2.4)	28.7 (1.4)	30.2 (1.3)	43.3 (3.2)	< 0.001^†^	31.1 (0.9)

Data are presented as percentages (SE) or means (SE)

^*^P value for differences between groups using the ×^2^ test

^†^*P* value for differences between groups using the one-way ANOVA

Table 3 shows the prevalence of periodontitis according to the CDC/AAP case definition. Prevalence of periodontitis was estimated to be 77.5% with 29.8% severe periodontitis, 22.7% moderate periodontitis and 22.3% mild periodontitis. The prevalence increased with age.

**Table 3:** Distribution of subjects according to the CDC/AAP case definition (Eke et al.2012) in total and according to age

Degree of periodontitis	Age Groups (years)						*P* Vallue^*^	Total
	20–34	35–44	45–54	55–64	65–74	75+		
No periodontitis	47 (50.0)	14 (19.4)	13 (21.3)	22 (16.7)	25 (14.1)	10 (22.7)	< 0.001	131 (22.5)
Mild periodontitis	27 (28.1)	31 (43.1)	25 (41.0)	30 (22.7)	31 (17.5)	3 (6.8)		147 (25.3)
Moderate periodontitis	21 (21.9)	18 (25.0)	13 (21.3)	38 (28.8)	36 (20.3)	6 (13.6)		132 (22.7)
Severe periodotitis	1 (1.0)	9 (12.5)	10 (16.4)	42 (31.8)	85 (48.0)	25 (56.8)		172 (29.6)

Data are presenterd as numbers (percentages)

^*^P value for differences between groups using the X^2^ test

**Fig. 2:**
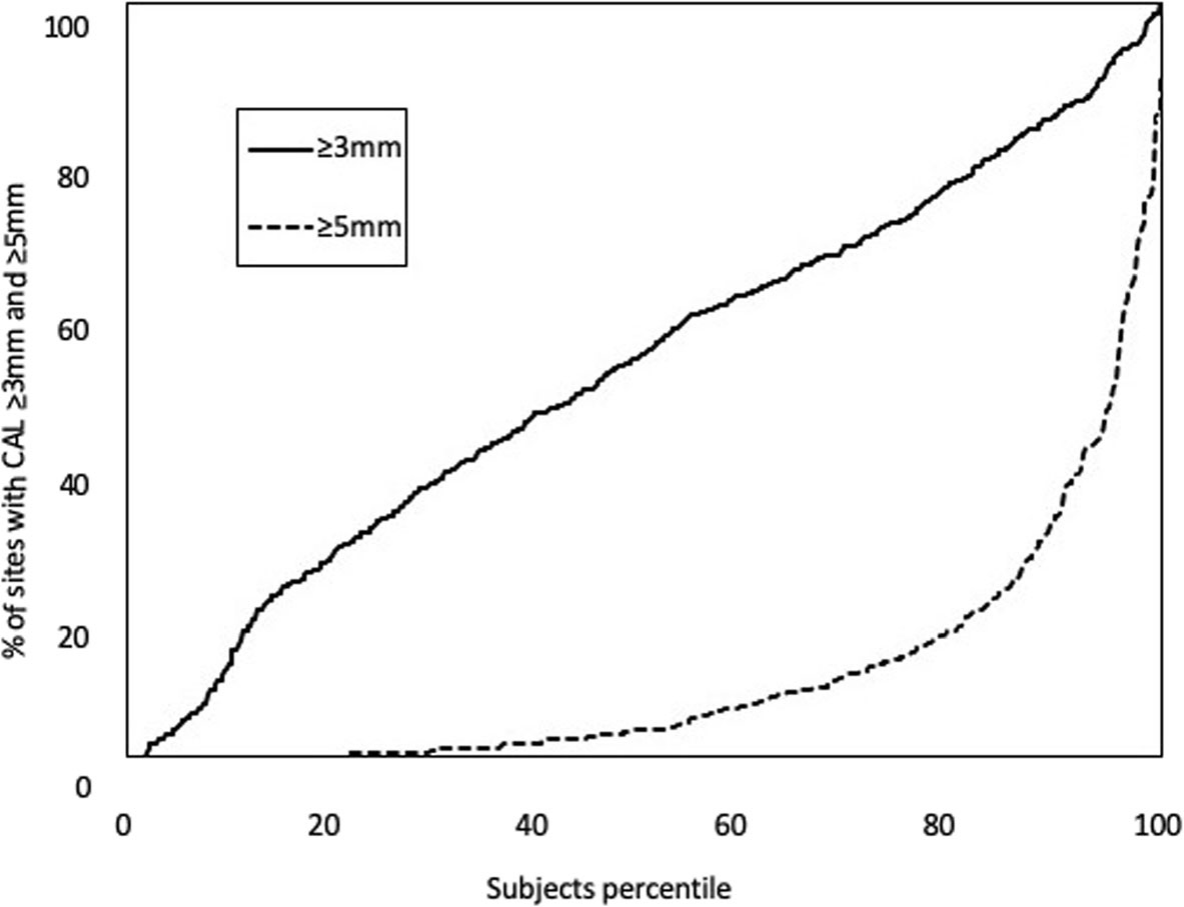
Percentile plots of cumulative attachment loss. The x-axis represents the subject percentile and the y-axis represents the percentage of sites in the subjects at or above thresholds of 3 and 5 mm

Figure 2 shows the percentile plots for all subjects at sites with CAL ≥3 mm and ≥ 5 mm. CAL ≥3 mm was evident in most patients and in 75.9% of these patients, its extent exceeded 30%. Clinical attachment loss of ≥5 mm was evident in 31.3% of participants, with the extent exceeding 10%; but for 10.9% of participants, it was ≥30%.

Table 4 presents the results of the mean percentage of CAL ≥5 mm in each age group by demographic, biologic and environmental characteristics. Significant differences were found in mean extent of attachment loss in some age groups by sex, smoking status, educational level, diabetes mellitus and plaque scores.

**Table 4:** The meean frequency of CAL ≥ 5 mm in each age grpuo of each characters

	Age Groups (years)						
	20-34	35-44	44-54	55-64	65-74	75+	Total
Gender							
female	1.0 (1.4**)**	2.9 (4.6)	12.4 (26.6)	13.6 (16.6)	23.6 (21.0)	24.9 (25.7)	9.6 (16.9)
male	0.8 (2.2)	3.4 (6.8)	6.9 (13.5)	9.4 (14.2)	16.7 (18.3)	19.8 (15.1)	15.0 (20.3)
*P*-value	0.81	0.74	0.43	0.22	0.02	0.46	
Smoking status							
Never smokers	0.6 (1.2)	3.7 (6.4)	6.8 (13.4)	9.9 (14.4)	17.4 (18.8)	16.8 (12.5)	10.3 (15.4)
Former and Current smokers	1.4 (2.9)	2.4 (5.2)	12,1 (24.7).	12.3 (16.0)	25.0 (20.8)	27.6( 24.3)	13.8 (20.0)
*P*-value	0.18	0.37	<0.01	0.46	0.02	0.08	
Educational levelLow							
Low and Middle	0.3 (0.7)	2.1 (2.5)	7.7 (22.2)	14.2 (20.5)	17.6 (18.6)	27.9 (14.0)	12.1 (16.9)
High	0.6 (1.0)	1.0 (1.0)	5.5 (12.0)	6.3 (7.5)	13.0 (13.5)	24.9 (30.0)	11.3 (13.9)
*P*-value	0.35	0,.29	0.73	0.04	0.48	0.89	
Body Mass Index							
<25 kg/m^2^	0.8 (2.0)	3.1 (5.7)	6.3 (12.3)	10.5 (15.2)	19.9 (19.3)	20.7 (21.2)	10.8 (16.4)
≥25 kg/m^2^	1.1 (1.8)	4.8 (9.2)	16.7 (30.8)	10.4 (13.9)	17.1 (19.7)	24.4 (16.2)	13.6 (19.0)
*P*-value	0.57	0.56	0.07	0.98	0.43	0.81	
Diabetes Melitus							
Yes			3.1(n=1)	6.4 (2.8	19.3 (20.0)	21.4 (19.8)	20.1 (17.5)
No			8.4 (18.1)	10.7 (15.1)	23.6 (17.7)	25.2 (22.2)	11.1(17.1)
*P*-value				0.53	0.41	0.73	
Tooth brushing Frequency							
<2 times/day	0.5 (0.8)	2.9 (5.0)	0.9 (1.1)	10.9 (9.4)	18.6 (12.3)	12.9(16.6)	11.6 (12.3)
≥2 times/day	0.9 (2.0)	3.2 (6.2)	9.8 (19.3)	10.0 (14.5)	19.8 (21.0)	24.2 (21.2)	11.3 (17.6)
*P*-value	0.34	0.9	0.15	0.83	0.75	0.13	
Use of inter-dental care device							
Yes	0.7 (1.3)	4.3 (8.1)	6.4 (14.1)	9.1 (13.9)	17.0 (18.0)	19.4 (17.8)	11.8 (18.1)
No	0.9 (2.2)	2.5 (4.0)	10.1 (20.6)	11.8 (15.7)	22.5 (21.2)	23.7 (21.5)	11.0 (15.8)
*P*-value	0.71	0.28	0.41	0.29	0.06	0.47	
Last Dental visit							
within last 12 months	0.2 (2.1)	3.3(6.6)	7.1 (14.1)	11.4 (15.6)	19.7 (21.0)	21.1 (19.4)	10.4 (16.6)
Less often	0.3 (1.3)	2.9 (3.8)	11.0 (23.9)	9.2 (13.9)	19.4 (18.4)	23.0 (21.2)	13.3 (18.0)
*P*-value	0.87	0.81	0.41	0.41	0.93	0.77	
Plaque score							
<20%	0.4 (1.0)	3.9 (3.4)	6.5 (16.5)	8.3 (13.8)	17.7 (20.1)		9.9 (16.2)
≥20%	1.0 (2,2)	2.8 (6.0)	9.2 (18.7)	11.8 (15.4)	20.5 (19.6)		12.1 (17.5)
*P*-value	<0.05	0.46	0.57	0.18	0.38		

blank: no subject was in one of the categories

## Discussion

The participants of this study were Japanese adults aged 20 to 89 years. The smoking rate, prevalence of diabetes, and proportion of participants with BMI > 25 kg/m^2^ were all lower than the mean values for the Japanese population as a whole. This suggests that the participants had good overall awareness of health issues [16].

The 2011 Survey of Dental Diseases [12] included an assessment of the community periodontal index (CPI) by partial inspection, which found that the prevalence of pockets of depth ≥ 4 mm ranged from 13.5% among individuals in their 20s to 50.8% among those in their 70s. The corresponding prevalence of pockets ≥6 mm in depth was 1.1 to 16.5%. These figures were clearly lower than those found in the present study. The difference between our results and those of the Survey of Dental Diseases may have stemmed from the use of partial inspection to assess the CPI, possibly causing some pockets to be overlooked. Baelum et al. [17] found that *Community Periodontal Index of Treatment Needs* data for ten teeth overestimated the prevalence and severity of attachment loss among younger people and underestimated it for those over 35 years of age. The fact that all teeth were covered by our study was thus a major advantage.

The proportion of tooth surfaces exhibiting pockets was lower in younger age groups and higher in older ones. This indicated that even if pockets were apparent in younger people, they were restricted to a small area of the teeth.

The mean CAL was 1.8 mm to 3.6 mm, with a greater difference due to age than that apparent for PPD. Similarly, to PPD, the percentage of tooth surfaces with attachment loss was lower in younger than in older patients.

The percentile plots for all subjects of sites with CAL ≥3 mm showed that the line was almost straight. On the other hand, in the same figure of CAL ≥5 mm, the slope of the 10 to 20% subset showed a substantially higher percentage of affected sites. This means that a small proportion of subjects show higher susceptibility to periodontitis.

In our study, female sex, smoking status, educational level, diabetes mellitus and plaque scores in some age groups affected the extent of attachment loss. However, the influence of dental visits was not significant. This could be in part explained by the fact that most patients visit dental practices only when they feel there is a problem. Thus, dentists in this district performed mainly symptomatic therapy. The fact that the Japanese health insurance system only covers symptomatic treatments, not prevention, may also be a contributing factor.

A recently reported epidemiological survey on periodontal disease carried out in the United States from 2009 to 2010 [18] found that 47.2% of adults aged 30 years or over had periodontitis. The prevalence of CAL ≥3 mm was 85.9% and of CAL ≥5 mm, 43.4%. In all of these categories, the prevalence increased with advancing age. The results of that study suggested that the severity of periodontitis is associated with male sex, Mexican-American ethnicity, educational level, poverty, and smoking. An epidemiological survey of a rural area of Thailand, however, found that the mean proportion of tooth surfaces with attachment loss of ≥4 mm was 23.9% in subjects in their 30s and 63.9% in subjects in their 60s, while the mean proportion with pockets ≥4 mm in depth was 11.6 to 20.5% [19]. A study by Corraini et al. [4] carried out in an isolated population in Brazil from 2005 to 2006 found that CAL of ≥3 mm was present in 100% of participants, and attachment loss of ≥5 mm was evident in 100% of those aged 50 years or more. In adults, the extent of tooth surfaces with attachment loss of ≥5 mm was also high (2.0 to 43.6%). The data from Takahagi City included a higher prevalence and extent of attachment loss than that reported by Eke et al. and was also lower than the results of the South American and Southeast Asian studies. Considering the above-mentioned results and the present study, these differences may be related to factors including ethnicity, educational level, and oral hygiene.

Inter-operator variation must also be considered. In the studies performed in other countries, measurements were carried out by multiple investigators; but in the Takahagi City survey, all measurements were made by a single periodontist. This was a major advantage in terms of the reproducibility of measurements.

We attempted to compare the results of this study with those of an epidemiological survey of 319 residents of Ushiku City, Ibaraki Prefecture, that was carried out in 1980 [3, 14]. At that time, the population of Ushiku City was about 50,000. Only numerical data provided in those papers were analyzed. In the early study, the age of participants was between 20 to 79 years and they were classified into six age groups. For comparison, participants in the present study who were more than 79 years were excluded, and a total of 573 subjects were divided into six age groups in the same way as in the early study (Table 5). The mean number of missing teeth per participant among all the participants in the 1980s survey was 7.2 ± 6.3. This number had declined to 6.1 ± 4.6 missing teeth in our survey (data not shown). In the 1980s survey, the mean number of missing teeth recorded was around four teeth for participants in their 20s and a mean of 15 teeth for those over 60 years, whereas in this study, the numbers were 3.3 ± 1.6 teeth for participants in their 20s and 9.3 ± 6.3 teeth for those in their 70s (data not shown). This means that participants in older age groups had significantly more teeth than in 1980s subjects. The mean proportion of tooth surfaces harboring plaque was 64% ± 17% in the 1980s survey and 58.2% ± 20.2% in the present study. In the 1980s survey, the mean proportion of BOP in participants in their 20s was approximately 35%, when compared with approximately 60 to 65% for elderly participants. In the present study, the mean proportion of BOP in participants in their 20s was 33.2% ± 13.2%, as compared with a range of 30.9% ± 17.1 to 33.4% ± 17.5% for those over 60 years, indicating a particularly marked change among elderly participants. The records from the 1980s survey indicated that treatment had involved mainly symptomatic therapy and treatment of acute symptoms. However, in the recent data from Takahagi City, 23% of participants were undergoing regular dental checkups, indicating a higher awareness of dentistry and oral hygiene among the general public. In the 1980s, survey participants in their 20s and 30s had the lowest scores; but in the recent study, participants in their 20s and 30s and also those in their 70s exhibited poor oral hygiene. The rates of interdental cleaner use and of regular dental checkups were lower among participants in their 20s and 30s, and this may have been reflected in their high plaque scores. As with the plaque scores, the proportion of tooth surfaces exhibiting BOP was also greatest among participants in their 20s and 30s and those in their 70s.

**Table 5:** Mean probing pocket depth, mean clinical attachment levels and percentage of participants in each age group with one or more sites exhibiting attachment levels exceeding specific thresholds

	1980’s							Current						
	20–29	30–39	40–49	50–59	60–69	70–79	Total	20–29	30–39	40–49	50–59	60–69	70–79	Total
Number of subjects	53	51	95	52	38	30	319	52	84	63	87	170	117	573
CAL														
Prevalence CAL ≥3 mm	81	94	98	98	90^a^	96	83	80.8	99.1	100	100	100	100	92.1
Prevalence CAL ≥5 mm	19	31^a^	55	62	84^a^	82	52	13.5	40.5	62	65.6	80.8	95.5	70.3
Mean CAL (mm)	1.2 ± 0.6	1.5 ± 0.5^†^	2.1 ± 0.8^†^	2.7 ± 1.0	3.7 ± 1.3	3.7 ± 1.5	2.2 ± 1.2	1.6 ± 0.5	2.2 ± 0.6	2.5 ± 0.5	2.9 ± 1.1	3.0 ± 0.8	3.4 ± 1.0	2.8 ± 1.0
PPD measures														
Mean PPD (mm)	2.3 ± 0.5	2.4 ± 0.4	2.6 ± 0.5	2.7 ± 0.5	2.8 ± 0.7	2.9 ± 0.8	2.5 ± 0.6	2.3 ± 0.2	2.4 ± 0.3	2.4 ± 0.4	2.5 ± 0.5	2.5 ± 0.5	2.7 ± 0.6	2.5 ± 0.5

^a^significant difference between 1980’s and current data at each of age group using x^2^ test

^†^significant difference between 1980’s and current data at each of age group using 95% confidence interval

The mean PPD was between 2.2 ± 0.5 mm and 2.8 ± 0.7 mm in the 1980s survey, as compared with between 2.3 ± 0.2 mm and 2.7 ± 0.6 mm in the present study. When the data were compared with a 95% confidence interval, there were no significant differences in the mean PPD among the two data sets. Despite the higher number of remaining teeth when compared with the 1980s, there was no change in the mean PPD in all age groups. BOP actually decreased, suggesting that some participants had undergone periodontal treatment and this may have helped preserve a large number of teeth with attachment loss. Ushiku City, from where the 1980s data were obtained, had at that time a population of around 50,000; the population has grown to approximately 80,000 today. The difference in size of the two cities may have had some effect.

The mean CAL was between 1.2 ± 0.6 and 3.7 ± 1.5 mm in the 1980s survey, as compared with between 1.6 ± 0.5 mm and 3.4 ± 1.0 mm in the recent study. The differences between the 1980s data and recent data were statistically significant for participants in their 30s and 40s. For the 30s group, the prevalence of attachment loss of ≥5 mm was significantly higher in the recent study when compared by chi-squared test (*p* < 0.05). The reason for this difference in mean CAL among relatively young age groups in recent data and data from the 1980s may lie in inter-operator variation in the measurement of CAL when the cement–enamel junction is below the gingival margin. In any case, the PPD for participants in their 20s and 30s was below the mean PPD, indicating the absence of root exposure in most cases, and within this range a high value of CAL does not necessarily indicate a pathological condition.

A lower prevalence of CAL of ≥5 mm in current participants compared with the 1980s data was seen only in participants in their 60s. Interestingly, in the current data, mean plaque score was the lowest in participants in their 40s and second-lowest in participants in their 50s. It may be speculated that the inhabitants in these age groups generally started to be more aware of oral hygiene.

Studies have compared the prevalence of periodontal disease in the 1970s and 1980s with that in recent years in several different countries [9–11, 20]. Although the parameters and diagnostic criteria used in these studies vary, oral hygiene and the prevalence of periodontal disease have improved in most countries in the twenty-first century compared with the 1970s and 1980s. Interestingly, however, the prevalence of severe periodontitis has not necessarily gone down. In a Swedish study, Hugoson et al. [9] classified participants into five categories according to the severity of periodontitis on clinical examination and radiographic findings. Between 1973 and 2003, the proportion of individuals with healthy gingival tissue who were categorized as Group 1 increased from 8 to 44%, and the prevalence of gingivitis and moderate periodontitis declined. However, there was no change between 1983 and 2003 in the proportion of individuals in Group 5, the most severe category, with periodontitis with significant bone resorption in at least two-thirds of tooth roots. Similarly, a Norwegian study by Skudutyte-Rysstad et al. [20] in patients over 35 years of age also found that although the number of participants with little or no bone loss decreased between 1973 and 2003, the proportion of participants in the most severe category (bone loss of over 20%) had hardly changed (from 6 to 7%). Periodontitis may thus be difficult to prevent by means of normal oral cleaning in highly susceptible individuals. The reason for the tendency for improvements in plaque score and gingival inflammation when compared with the 1980s, although the frequency of attachment loss actually increased, may have been the presence of individuals who were highly susceptible to periodontitis.

## Conclusion

In conclusion, our survey in Takahagi City, Ibaraki Prefecture found that although mild periodontal pockets and attachment loss were present in almost all age groups, the prevalence and extent of deep periodontal pockets and moderate or worse attachment loss increased with advancing age. Periodontal disease was still prevalent in the current Japanese population, even though some improvement occurred since the 1980s. Proper public health programs thus need to be established.

## Data Availability

The datasets used and analyzed during the current study are available from the corresponding author on reasonable request.

## Abbreviations

BMI: Body mass index
BOP: Bleeding on probing
CAL: Clinical attachment level
CDC/AAP: Centers Of Disease Control And Prevention/American Academy Of Periodontology
CPI: Community periodontal index
PPD: Probing pocket depth
SD: Standard deviation
SE: Standard error

## References

[1] Baelum V, Scheutz F (2000). Periodontal diseases in Africa. Periodontol.

[2] Corbet EF, Leung WK (2000). Epidemiology of periodontitis in the Asia and Oceania regions. Periodontol.

[3] Dye BA (2000). Global periodontal disease epidemiology. Periodontol.

[4] Corraini P, Baelum V, Pannuti CM, Pustiglioni AN, Romito GA, Pustiglioni FE (2008). Periodontal attachment loss in an untreated isolated population of Brazil. J Periodontol.

[5] Holtfreter B, Schwahn C, Biffar R, Kocher T (2009). Epidemiology of periodontal diseases in the study of health in Pomerania. J Clin Periodontol.

[6] Montero-Aguilar M, Muñoz-Torres F, Elías-Boneta AR, Dye B, Joshipura KJ (2012). High levels of periodontal disease among the older adult population in San Juan, Puerto Rico. Commun Dent Health.

[7] Chrysanthakopoulos NA (2012). Periodontal disease status in an isolated Greek adult population. J Dent (Teheran).

[8] Figueiredo A, Soares S, Lopes H, dos Santos JN, Ramalho LM, Cangussu MC, Cury PR (2013). Destructive periodontal disease in adult Indians from Northeast Brazil: cross-sectional study of prevalence and risk indicators. J Clin Periodontol.

[9] Hugoson A, Sjödin B, Norderyd O (2008). Trends over 30 years, 1973-2003, in the prevalence and severity of periodontal disease. J Clin Periodontol.

[10] Borrell LN, Talih M (2012). Examining periodontal disease disparities among U.S. adults 20 years of age and older: NHANES III (1988-1994) and NHANES 1999-2004. Public Health Rep.

[11] Haisman-Welsh RJ, Thomson WM (2012). Changes in periodontitis prevalence over two decades in New Zealand: evidence from the 1988 and 2009 national surveys. NZ Dent J.

[12] Statistical tables of the survey of dental diseases (2011) part 1 .The Ministry of Health, Labour and Welfare home page. http://www.mhlw.go.jp/toukei/list/dl/62-17c23-1.pdf.Accessed in 2011.

[13] Okamoto H, Yoneyama T, Lindhe J, Haffajee A, Socransky S (1988). Methods of evaluating periodontal disease data in epidemiological research. J Clin Periodontol.

[14] Yoneyama T, Okamoto H, Lindhe J, Socransky SS, Haffajee AD (1988). Probing depth, attachment loss and gingival recession. Findings from a clinical examination in Ushiku, Japan. J Clin Periodontol.

[15] Silness J, Loe H. Periodontal disease in pregnancy. II. Correlation between hygiene and periodontal condition. Acta Odontol Scand. 1964:121–35. 10.3109/00016356408993968 14158464

[16] Baelum V, Manji F, Wanzala P, Fejerskov O (1995). Relationship between CPITN and periodontal attachment loss findings in an adult population. J Clin Periodontol.

[17] Eke PI, Dye BA, Wei L, Thornton-Evans GO, Genco RJ (2012). CDC periodontal disease surveillance workgroup: James Beck (University of North Carolina, Chapel Hill, USA), Gordon Douglass (past president, American Academy of periodontology), Roy page (University of Washington). Prevalence of periodontitis in adults in the United States: 2009 and 2010. J Dent Res.

[18] Baelum V, Pisuithanakan S, Teanpaisan R, Pithpornchaiyakul W, Pongpaisal S, Papapanou PN, Dahlén G, Fejerskov O (2003). Periodontal conditions among adults in southern Thailand. J Periodontal Res.

[19] Skudutyte-Rysstad R, Eriksen HM, Hansen BF (2007). Trends in periodontal health among 35-year-olds in Oslo, 1973-2003. J Clin Periodontol.

[20] National health and nutrition survey in 2010. The Ministry of Health, Labour and Welfare home page. https://www.mhlw.go.jp/stf/houdou/2r9852000000xtwq.html

